# Pharmacokinetics of Nirmatrelvir and Ritonavir in COVID-19 Patients with End-Stage Renal Disease on Intermittent Hemodialysis

**DOI:** 10.1128/aac.01229-22

**Published:** 2022-10-26

**Authors:** Tilman Lingscheid, Martina Kinzig, Anne Krüger, Nils Müller, Georg Bölke, Pinkus Tober-Lau, Friederike Münn, Helene Kriedemann, Martin Witzenrath, Leif E. Sander, Fritz Sörgel, Florian Kurth

**Affiliations:** a Department of Infectious Diseases and Respiratory Medicine, Charité–Universitätsmedizin Berlin, corporate member of Freie Universität Berlin and Humboldt-Universität zu Berlin, Berlin, Germany; b IBMP–Institute for Biomedical and Pharmaceutical Research, Nürnberg-Heroldsberg, Germany; c Department of Nephrology and Intensive Care, Charité–Universitätsmedizin Berlin, corporate member of Freie Universität Berlin and Humboldt-Universität zu Berlin, Berlin, Germany; d German Center for Lung Research, DZL, Berlin, Germany; e Berlin Institute of Health, Berlin, Germany; f Institute of Pharmacology, West German Heart and Vascular Centre, University of Duisburg-Essen, Essen, Germany; g Department of Tropical Medicine, Bernhard Nocht Institute for Tropical Medicine, Hamburg, Hamburg, Germany

**Keywords:** COVID-19, end-stage renal disease, hemodialysis, nirmatrelvir/ritonavir, Paxlovid

## Abstract

Nirmatrelvir/ritonavir is an effective antiviral therapy against severe acute respiratory syndrome coronavirus 2 (SARS-CoV-2). Use is not recommended in patients with end-stage renal disease (ESDR) due to a lack of data. We investigated the pharmacokinetics of nirmatrelvir/ritonavir (150 mg/100 mg twice a day) in four patients with ESRD undergoing hemodialysis. Nirmatrelvir peak concentrations ranged from 4,563 to 7,898 ng/mL and declined after hemodialysis. Concentrations were up to 4-fold higher but still within the range known from patients without ESRD, without accumulation of nirmatrelvir after the end of treatment.

## INTRODUCTION

Nirmatrelvir/ritonavir (N/r) is highly effective for early treatment of high-risk patients with severe acute respiratory syndrome coronavirus 2 (SARS-CoV-2) infection (1, 2). End-stage renal disease (ESRD) is a major risk factor for severe COVID-19. Moreover, immunogenicity and effectiveness of COVID-19 vaccines is reduced in patients with ESRD (3), making them an important target group for preventive treatment. Nirmatrelvir is rapidly metabolized via CYP3A4 when given alone. In combination with ritonavir, almost no metabolic breakdown of nirmatrelvir takes place, and the drug is excreted primarily through renal elimination (4). In patients with moderate renal impairment (estimated glomerular filtration rate [eGFR], 31 to 59 mL/min), dose adjustment is recommended from 300 mg/100 mg twice a day (BID) to 150 mg/100 mg BID. In patients with severe renal impairment (eGFR < 30 mL/min) undergoing hemodialysis (HD), use of N/r is currently not recommended due to insufficient data (5–7). A recent case series investigated a dose of 300 mg/100 mg N/r on day 1, followed by 150 mg/100 mg once daily in ESRD patients, and showed good tolerance but without pharmacokinetic measurements (8, 9). Pharmacokinetic assessment of N/r in patients with ESRD on chronic intermittent HD is therefore of particular interest for clinicians (7, 10, 11). Herein, we report data on pharmacokinetics and hepatic tolerance of nirmatrelvir and ritonavir treatment in four ESRD patients on intermittent HD.

Four patients undergoing outpatient HD therapy at our center tested positive for SARS-CoV-2 in 2022. Based on the individual risk profile, nephrologists initiated antiviral therapy with N/r. Treatment was started 3 to 5 days after the first positive SARS-CoV-2 reverse transcription (RT)-PCR test. All patients were instructed to self-administer the recommended reduced dose for renal impairment of 150 mg/100 mg N/r BID orally at 7 a.m. and 7 p.m. for 5 days. Patient 1 was on constant ritonavir therapy (100 mg once daily), due to a well controlled HIV infection. The patient characteristics are shown in the supplemental material. In patients 1, 2, and 3, HD started in the morning 2 to 3 h after intake of the medication. In patient 4, HD started approximately 6 h after the morning dose.

Blood sampling was performed as part of the clinical routine within the framework of an observational study rather than sampling within an interventional trial. The details of dialysis are shown in the supplemental material. In short, blood-flow rates were 250 to 300 mL/min with dialysate flow rates of 500 mL/min for all patients with various ultrafiltration rates. Blood samples (plasma and serum) were drawn at the beginning and the end of each HD. Samples of postfilter dialysate were drawn at 0.5, 2, and 4 h after start of HD.

N/r concentrations were quantified in human plasma, serum, and dialysate by liquid chromatography with tandem mass spectrometry (LC-MS/MS). Assay details are shown in the supplemental material.

The described patients are part of the Pa-COVID-19 cohort study, a prospective observational study conducted at Charité–Universitätsmedizin Berlin (12). Written informed consent was provided by all patients prior to inclusion, and all patients of the ongoing cohort with N/r treatment and ESRD are included in this report. The study was approved by the ethics committee of Charité–Universitätsmedizin Berlin (approval EA2/066/20), conducted according to the Declaration of Helsinki and Good Clinical Practice principles (ICH 1996), and registered in the German and WHO international clinical trials registry (DRKS00021688).

**FIG 1:**
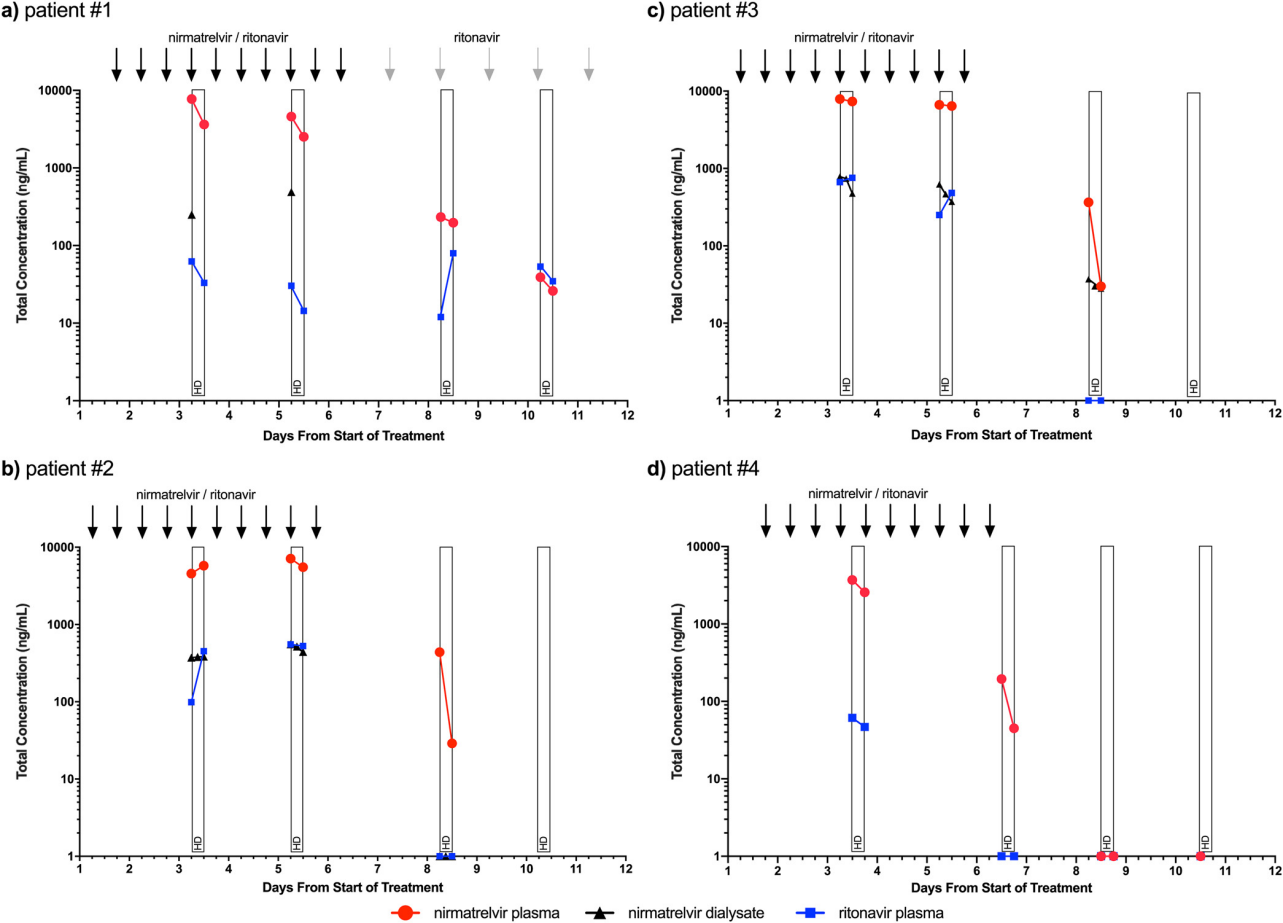
Pharmacokinetics of nirmatrelvir in plasma (red circles), dialysate (black triangles), and ritonavir in plasma (blue squares) sampled pre- and posthemodialysis (HD) during and after the 5-day course of nirmatrelvir/ritonavir (days 1 to 5). (a) Patient 1. (b) Patient 2. (c) Patient 3. (d) Patient 4. Concentrations of the lower limit of quantification are positioned on the *x* axis, not suggesting that there is a measurable level but in order to demonstrate zero results on the logarithmic scale of the *y* axis. Each thick black arrow resembles one dose of nirmatrelvir/ritonavir (150 mg/100 mg); gray arrows represent one dose of ritonavir (100 mg) in patient 1. Pre-HD sample of patient 1 on day 3 is a serum sample instead of plasma.

The treatment courses, HD sessions, and pharmacokinetic measurements of nirmatrelvir and ritonavir in plasma and dialysate of individual patients are shown in Fig. 1. Patient 1 presented with a peak nirmatrelvir concentration of 7,745 ng/mL 2 h after intake of the fourth dose of N/r. During the 4-h HD, plasma concentration fell by 53% to 3,636 ng/mL. At the second sampling on day 5, a peak plasma concentration of 4,601 ng/mL was measured, which fell by 45% to 2,518 ng/mL post-HD. Both patients 2 and 3 received HD 3 days after intake of the fifth dose of N/r. Nirmatrelvir levels of patient 2 increased during HD from 4,563 to 5,765 ng/mL; patient 3 showed stable concentrations of 7,898 and 7,345 ng/mL pre- and post-HD, respectively. Plasma levels on treatment day 5 were 7,116 ng/mL pre-HD and 5,521 ng/mL post-HD in patient 2 and 6,653 ng/mL pre-HD and 6,417 ng/mL post-HD in patient 3. Measurements on day 8 (3 days after end of treatment) showed levels of 438 and 365 ng/mL pre-HD with a decline to 29 and 30 ng/mL in patients 2 and 3, respectively. Patient 4 presented with a nirmatrelvir plasma concentration of 3,704 ng/mL 6 h after intake of the fourth dose of N/r. Post-HD plasma concentration fell by 38% to 2,308 ng/mL at 11 to 12 h after drug intake. Measurement after the last dose on day 6 showed concentrations of 187 ng/mL and 47 ng/mL pre- and post-HD, respectively. Further measurement showed nirmatrelvir levels below the lower limit of quantification, likewise to negative ritonavir levels.

Nirmatrelvir concentrations were measured in plasma and serum with a mean accuracy between 83 and 109%. Plasma and serum levels showed the same value, which may be important for later studies when only one sampling technique is used for reasons of simplicity. Detailed individual measurements are shown in the supplemental material (Table S1). Levels of nirmatrelvir in dialysate ranged from 783 ng/mL to levels below the lower limit of quantification, with higher concentrations in plasma associated with higher concentrations in dialysate (Table S2). Ritonavir plasma levels varied to a very high extent between the different patients with measurements ranging from 14 to 756 ng/mL (Table S3). Overall, no signs of relevant drug-related toxicity or hepatic impairment were observed (Fig. S1). No patient reported severe adverse events attributed to the medication.

All patients had a moderate course of COVID-19. Patient 1 was tested negative for SARS-CoV-2 on day 10. Patient 2 had a low viral load 7 days after initial diagnosis and remained positive for 2 weeks. Patient 3 had low viral load after 10 days and remained positive for 3 weeks. Patient 4 remained positive for 6 weeks. Initial viral load from nasopharyngeal swabs was high (*C_t_* value <30), initially decreased (*C_t_* -value >30), spiked again 5 days after end of treatment, and remained positive with a low viral load for 4 weeks.

Measured peak plasma concentrations of nirmatrelvir were up to 4-fold higher than median levels of patients with normal kidney function and full N/r dose, which ranged around 2,210 ng/mL (5). The 95th predicted nirmatrelvir *C*_max_ on day 5 of treatment is assumed to be 10,000 ng/mL among patients without renal impairment as stated in the U.S. Food and Drug Administration (FDA)’s review (5); this level was not reached at peak measurements 2 to 3 h after drug intake in our patients. Despite elevated nirmatrelvir levels, no accumulation of nirmatrelvir was observed, and no safety concerns arose from elevated liver function tests or adverse events. Exact *C*_min_ levels were not determined in our study as the HD sessions of the majority of patients ran in the morning soon after drug intake; the post-HD sample of patient 4, however, corresponds well to *C*_min_ as it was sampled approximately 11 h after drug intake, 1 h before intake of the evening dose of N/r. The respective nirmatrelvir concentration of 2,308 ng/mL is within the 90% prediction interval at steady state yet clearly above the 90% inhibitory concentration (IC_90_) of 292 ng/mL of nirmatrelvir for SARS-CoV-2 (5, 7). After the end of treatment, nirmatrelvir levels declined rapidly but were still measurable 3 days after treatment, whereas ritonavir levels were not measurable. Nirmatrelvir plasma levels pre-HD on day 8 still partly reached the IC_90_ (5, 7). Thus, antiviral exposure to nirmatrelvir may be longer in patients with ESRD, which may be a welcome effect in light of the debate of relapses after N/r therapy and proposed longer treatment phases (13). In contrast, patient 4 had subtherapeutic nirmatrelvir levels on day 4 of treatment. Whether this caused the prolonged virus replication in the patient remains speculative.

Ritonavir plasma levels were highly variable but in general within the range of data published earlier (14, 15). Nevertheless, the high nirmatrelvir levels indicate adequate ritonavir exposure in spite of variable plasma concentrations. Our study is limited by fixed sampling time points, making calculations of the area under the concentration-time curve (AUC), extraction rate, and sieving coefficient not possible.

In summary, our data suggest that use of N/r in patients with ESRD and HD results in peak plasma concentrations at the higher end of levels observed in patients without ESRD. However, no accumulation of nirmatrelvir was observed, and plasma levels declined rapidly within a few days after end of treatment.

## References

[1] Hammond J, Leister-Tebbe H, Gardner A, Abreu P, Bao W, Wisemandle W, Baniecki M, Hendrick VM, Damle B, Simón-Campos A, Pypstra R, Rusnak JM, EPIC-HR Investigators. 2022. Oral nirmatrelvir for high-risk, nonhospitalized adults with COVID-19. N Engl J Med 386:1397–1408. 10.1056/NEJMoa2118542. 35172054 PMC8908851

[2] Najjar-Debbiny R, Gronich N, Weber G, Khoury J, Amar M, Stein N, Goldstein LH, Saliba W. 2022. Effectiveness of Paxlovid in reducing severe COVID-19 and mortality in high risk patients. Clin Infect Dis ciac443. 10.1093/cid/ciac443. 35653428 PMC9214014

[3] Schrezenmeier E, Bergfeld L, Hillus D, Lippert JD, Weber U, Tober-Lau P, Landgraf I, Schwarz T, Kappert K, Stefanski AL, Sattler A, Kotsch K, Dörner T, Sander LE, Budde K, Halleck F, Kurth F, Corman VM, Choi M. 2021. Immunogenicity of COVID-19 tozinameran vaccination in patients on chronic dialysis. Front Immunol 12:690698. 10.3389/fimmu.2021.690698. 34276681 PMC8284337

[4] Owen DR, Allerton CMN, Anderson AS, Aschenbrenner L, Avery M, Berritt S, Boras B, Cardin RD, Carlo A, Coffman KJ, Dantonio A, Di L, Eng H, Ferre R, Gajiwala KS, Gibson SA, Greasley SE, Hurst BL, Kadar EP, Kalgutkar AS, Lee JC, Lee J, Liu W, Mason SW, Noell S, Novak JJ, Obach RS, Ogilvie K, Patel NC, Pettersson M, Rai DK, Reese MR, Sammons MF, Sathish JG, Singh RSP, Steppan CM, Stewart AE, Tuttle JB, Updyke L, Verhoest PR, Wei L, Yang Q, Zhu Y. 2021. An oral SARS-CoV-2 Mpro inhibitor clinical candidate for the treatment of COVID-19. Science 374:1586–1593. 10.1126/science.abl4784. 34726479

[5] Center for Drug Evaluation and Research Review. 2021. Emergency use authorization for Paxlovid. https://www.fda.gov/media/155194/download.

[6] European Medicines Agency. 2022. Paxlovid: EPAR—product information: summary of product characteristics. https://www.ema.europa.eu/en/documents/product-information/paxlovid-epar-product-information_en.pdf.

[7] European Medicines Agency: Committee for Medicinal Products for Human Use (CHMP): 2022. Assessment report: Paxlovid. https://www.ema.europa.eu/en/documents/assessment-report/paxlovid-epar-public-assessment-report_en.pdf.

[8] Hiremath S, McGuinty M, Argyropoulos C, Brimble KS, Brown PA, Chagla Z, Cooper R, Hoar S, Juurlink D, Treleaven D, Walsh M, Yeung A, Blake P. 2022. Prescribing nirmatrelvir/ritonavir for COVID-19 in advanced CKD. Clin J Am Soc Nephrol 17:1247–1250. 10.2215/CJN.05270522. 35680135 PMC9435977

[9] Brown PA, McGuinty M, Argyropoulos C, Clark EG, Colantonio D, Giguere P, Hiremath S. 2022. Early experience with modified dose nirmatrelvir/ritonavir in dialysis patients with coronavirus disease-2019. medRxiv 10.1101/2022.05.18.22275234. PMC10103226 36723285

[10] Eng H, Dantonio AL, Kadar EP, Obach RS, Di L, Lin J, Patel NC, Boras B, Walker GS, Novak JJ, Kimoto E, Singh RSP, Kalgutkar AS. 2022. Disposition of nirmatrelvir, an orally bioavailable inhibitor of SARS-CoV-2 3C-like protease, across animals and humans. Drug Metab Dispos 50:576–590. 10.1124/dmd.121.000801. 35153195

[11] Saravolatz LD, Depcinski S, Sharma M. 2022. Molnupiravir and nirmatrelvir-ritonavir: oral COVID antiviral drugs. Clin Infect Dis ciac180. 10.1093/cid/ciac180. 35245942 PMC9383515

[12] Kurth F, Roennefarth M, Thibeault C, Corman VM, Müller-Redetzky H, Mittermaier M, Ruwwe-Glösenkamp C, Heim KM, Krannich A, Zvorc S, Schmidt S, Kretzler L, Dang-Heine C, Rose M, Hummel M, Hocke A, Hübner RH, Opitz B, Mall MA, Röhmel J, Landmesser U, Pieske B, Knauss S, Endres M, Spranger J, Mockenhaupt FP, Tacke F, Treskatsch S, Angermair S, Siegmund B, Spies C, Weber-Carstens S, Eckardt KU, Schürmann D, Uhrig A, Stegemann MS, Zoller T, Drosten C, Suttorp N, Witzenrath M, Hippenstiel S, von Kalle C, Sander LE. 2020. Studying the pathophysiology of coronavirus disease 2019: a protocol for the Berlin prospective COVID-19 patient cohort (Pa-COVID-19). Infection 48:619–626. 10.1007/s15010-020-01464-x. 32535877 PMC7293426

[13] Coulson JM, Adams A, Gray LA, Evans A. 2022. COVID-19 “Rebound” associated with nirmatrelvir/ritonavir pre-hospital therapy. J Infect 85:436–480. 10.1016/j.jinf.2022.06.011. PMC9212499 35718206

[14] Das Mishra T, Kurani H, Singhal P, Shrivastav PS. 2012. Simultaneous quantitation of HIV-protease inhibitors ritonavir, lopinavir and indinavir in human plasma by UPLC-ESI-MS-MS. J Chromatogr Sci 50:625–635. 10.1093/chromsci/bms048. 22562821

[15] Gatti G, Di Biagio A, Casazza R, De Pascalis C, Bassetti M, Cruciani M, Vella S, Bassetti D. 1999. The relationship between ritonavir plasma levels and side-effects: implications for therapeutic drug monitoring. AIDS 13:2083–2089. 10.1097/00002030-199910220-00011. 10546861

