# Supplementary material for: Pharmacokinetics of Nirmatrelvir and Ritonavir in COVID-19 Patients with End-Stage Renal Disease on Intermittent Hemodialysis
Source: Antimicrob Agents Chemother. 2022 Oct 26;66(11):e01229-22. doi: 10.1128/aac.01229-22 (PMC9664846; doi:10.1128/aac.01229-22)
Supplement: Supplemental file 1 — Supplemental material. Download aac.01229-22-s0001.pdf, PDF file, 0.7 MB [file aac.01229-22-s0001.pdf]

Supplementary material:

Patients:

Patient #1: age: late sixties; male.

Relevant medical history: diabetic nephropathy, intermittent hemodialysis for four years, no residual renal function; diabetes mellitus type 2, COPD GOLD IV, peripheral arterial disease, coronary artery disease, HIV since 10 years.

Relevant medication: aspirin 100mg od, insulin varying doses, fluvastatin 40mg od (paused), clopidogrel 75mg od (paused), telmisartan 40mg od, cinacalcet 30mg od, raltegravir 400mg bid, lamivudine 100mg od, darunavir 800mg od, ritonavir 100mg od.

Patient #2: age: early sixties; male.

Relevant medical history: hypertensive nephropathy, intermittent hemodialysis for three years, no residual renal function coronary artery disease, peripheral arterial disease diabetes mellitus type 2.

Relevant medication: aspirin 100mg od, atorvastatin 40mg od paused, l-thyroxin 50µg od, pantoprazole 40mg od, tilidin 100mg bd, varying insulin doses,

Patient #3: age: late seventies; male.

Relevant medical history: diabetic nephropathy, intermittent hemodialysis for two years, no residual renal function, liver cirrhosis CHILD B, diabetes mellitus type 2, coronary artery disease, atrial fibrillation.

Relevant medication: aspirin 100mg od, simvastatin 20mg od (paused), pantoprazole 20mg od, pregabalin 25mg od, semaglutide 1mg twice weekly, insulin varying doses, allopurinol 100mg od.

Patient #4: age: late sixties; male.

Relevant medical history: diabetic/hypertensive nephropathy, intermittent hemodialysis for two years, no residual renal function, diabetes mellitus type 2, coronary artery disease, atrial fibrillation.

Relevant medication: bisoprolol 5mg bid, insulin varying doses, pantoprazole 20mg bid, clopidogrel 75mg od (paused), pregabalin 25mg od, sevelamer 800mg tid.

Additional Methods:

Sampling: K_3-EDTA liquid blood collection tubes containing ethylenediaminetetraacetic acid tri potassium salt to prevent clotting and CAT serum separator tubes (Greiner Bio-One, Austria) were used and processed within a maximum of one hour. Plasma/serum was separated by centrifugation and stored at -80°C prior to pharmacokinetic analyses. Dialysate was collected in no additive tubes (Greiner Bio-One, Austria) aliquoted and stored likewise.

Hemodialysis:

HD was performed using the Artis Physio® (Baxter Deutschland GmbH, Unterschleißheim) dialysis machine with a high-flux dialyzer (Revaclear 400 ®, Baxter). Duration of each dialysis session was 240 minutes for patients #1, #2 and #3; 300 minutes for patient #4. In all cases venous access was established via double AV-fistula puncture. Blood-flow rates were 250-300 mL/min with dialysate flow rates of 500 mL/min for all patients. Net ultrafiltration rates of 500-600 mL/h for patient #1, 900-1100mL/h for patient #2, 750-1200mL/h for patient #3 and 800-900mL/h for patient #4 were achieved. Unfractionated heparin was given to patient #1, fractioned heparin to patient #2, whereas in patients #3 and #4 a citrate anticoagulation protocol was used.

Determination of nirmatrelvir and ritonavir by liquid chromatography with tandem mass spectrometry (LC-MS/MS):

25µL of each sample was deproteinized with 150µL acetonitrile (containing the internal standard piperacillin-d_5), subsequently vortex-shaken and centrifuged. The supernatant was further diluted with Milli-Q® water and 10µL of each sample was injected into the LC-MS/MS system. The chromatographic system consisted of a binary LC-pump (Agilent 1200 Series, Agilent Technologies, Waldbronn, Germany) and a CTC Combi Pal Autosampler (CTC Analytics, Zwingen, Switzerland) set to +4°C. The separation was achieved on a Ascentis® Express RP-Amid (Supelco®, Darmstadt, Germany) using gradient elution at ambient temperature with 0.1% (v/v) formic acid in water (A) and 0.1% (v/v) formic acid in acetonitrile. Initial conditions were 20% B at a flow rate of 900µL/min. Gradient: 0-1.0 min: 20 to 90% B, flow rate: 900 to 1200µL/min, 1.0-1.3 min: 90% B flow rate: 1200µL/min, 1.31-2.5 min: 90 to

20% B flow rate: 1200µL/min. The detection was performed using an SCIEX API 5500™ triple quadrupole mass spectrometer equipped with turbo ion spray interface (SCIEX, Concord, Ontario, Canada). High purity nitrogen gas was used as nebulizer, curtain, auxiliary and collision gas. The spectrometer was operated in positive ion mode for the detection of nirmatrelvir, ritonavir and the internal standard in human plasma, serum and dialysate samples with the following parameters: curtain gas (CUR): 45.0psi, ion spray voltage (IS): 5000.0V, source temperature (TEM): 500.0°C, Auxiliary Gas 1 and Gas 2 (GS1 and GS2): 60.0psi and 70.0psi, CAD: medium, entrance potential (EP): 10.0V, cell exit potential (CXP): 17.0V. Quantification was performed using multiple reaction monitoring (MRM) with the conditions and transitions for nirmatrelvir (500.5→402.0, DP 150, CE 22), ritonavir (721.3→296.1, DP 100, CE 25) and the internal standard (523.2→148.4, DP 80, CE 35). The pause time was set to 5msec. Under these conditions nirmatrelvir, ritonavir and the internal standard were eluted after 1.7 min, 1.9 min, 1.2 min, 1.6 min, respectively. Data acquisition and processing of raw data was performed using Analyst software version 1.6.2 (SCIEX, Concord, Ontario, Canada).

Quantification of N/r was performed by peak area ratio of analytes to internal standard. The linearity of the calibration curve for nirmatrelvir was proven from 14.69 to 4753ng/mL and for ritonavir from 10.87 to 3516ng/mL. The lower limit of quantification in all matrices was set to 14.69ng/mL for nirmatrelvir and to 10.87ng/mL for ritonavir. No interferences in the chromatogram were observed for nirmatrelvir and ritonavir and the internal standard in human plasma, serum, and liquor. The intra-and inter-day precision of the spiked quality control samples was below 5% with an intra-and inter-day accuracy between 90% and 110%.

Figure S1: Monitoring of Liver Functioning Tests on Hemodialysis Days.

A: Patient #1

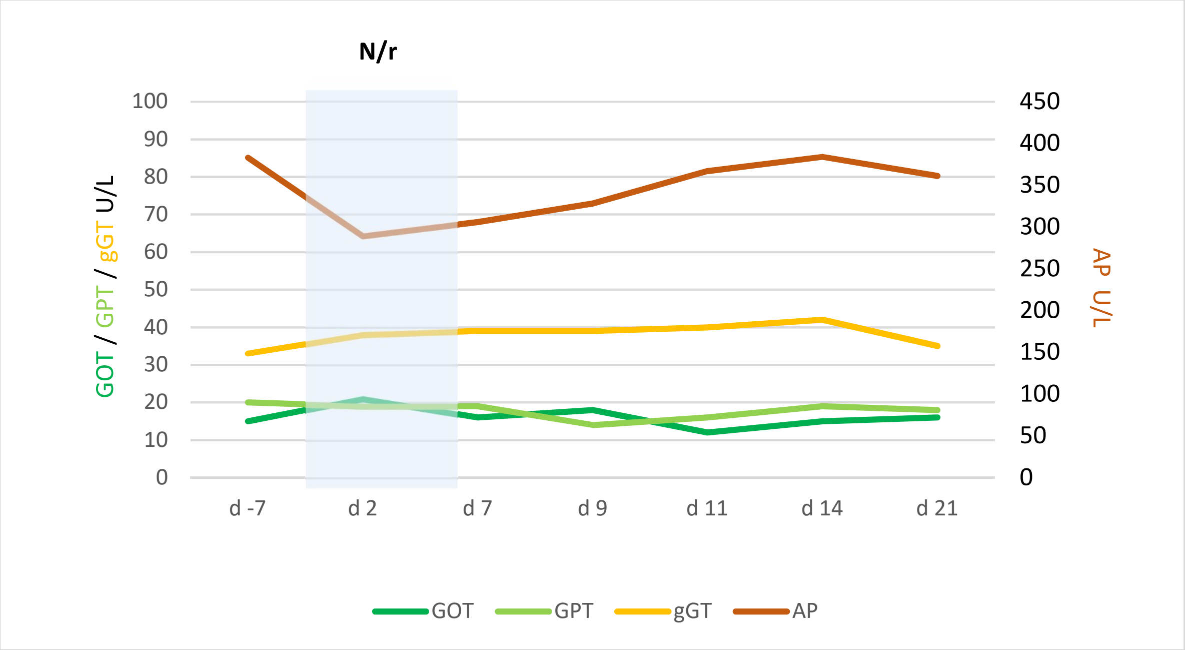

B: Patient #2

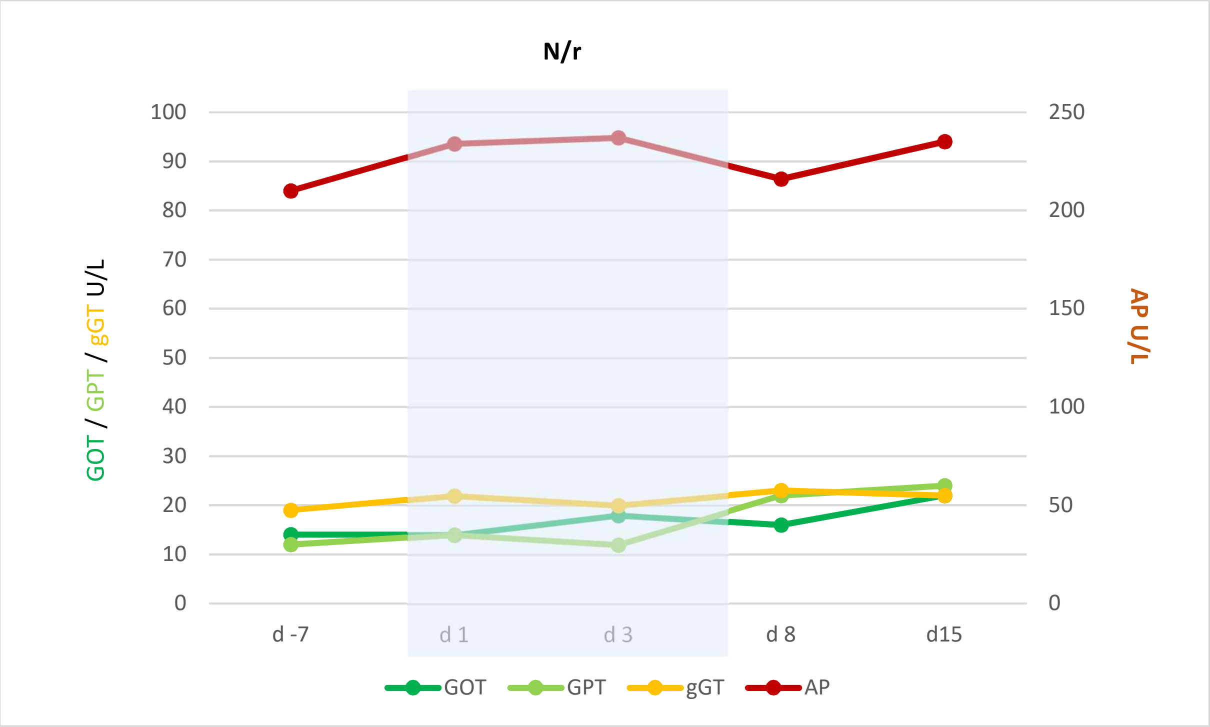

C: Patient #3

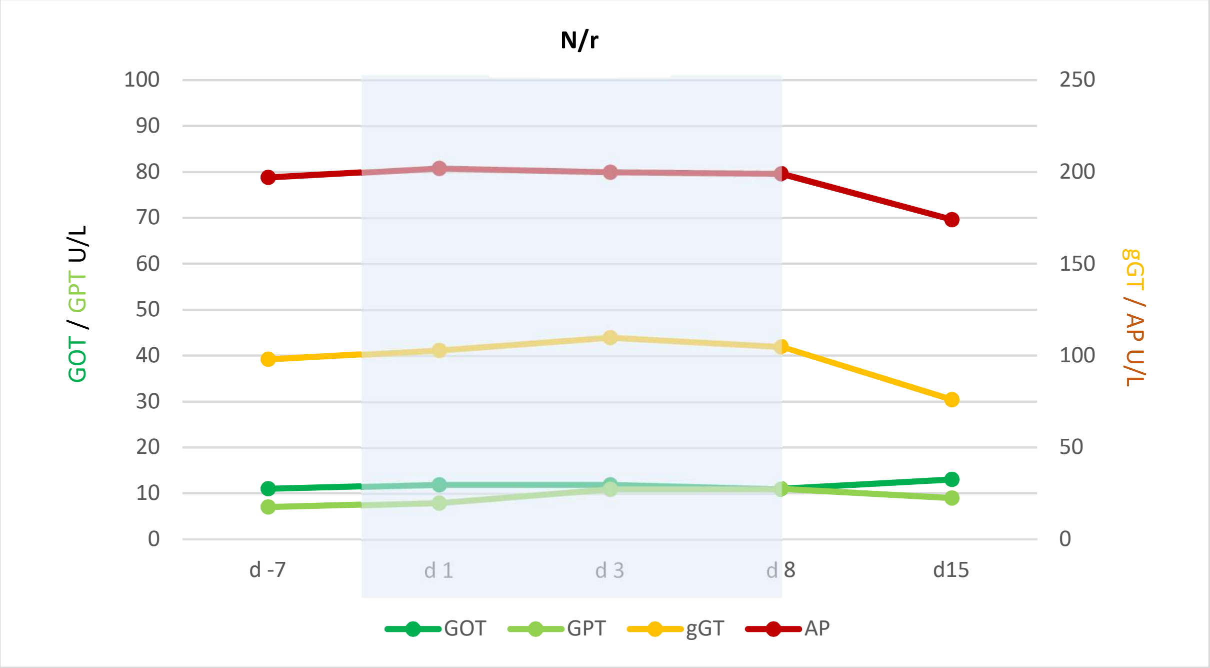

D: Patient #4

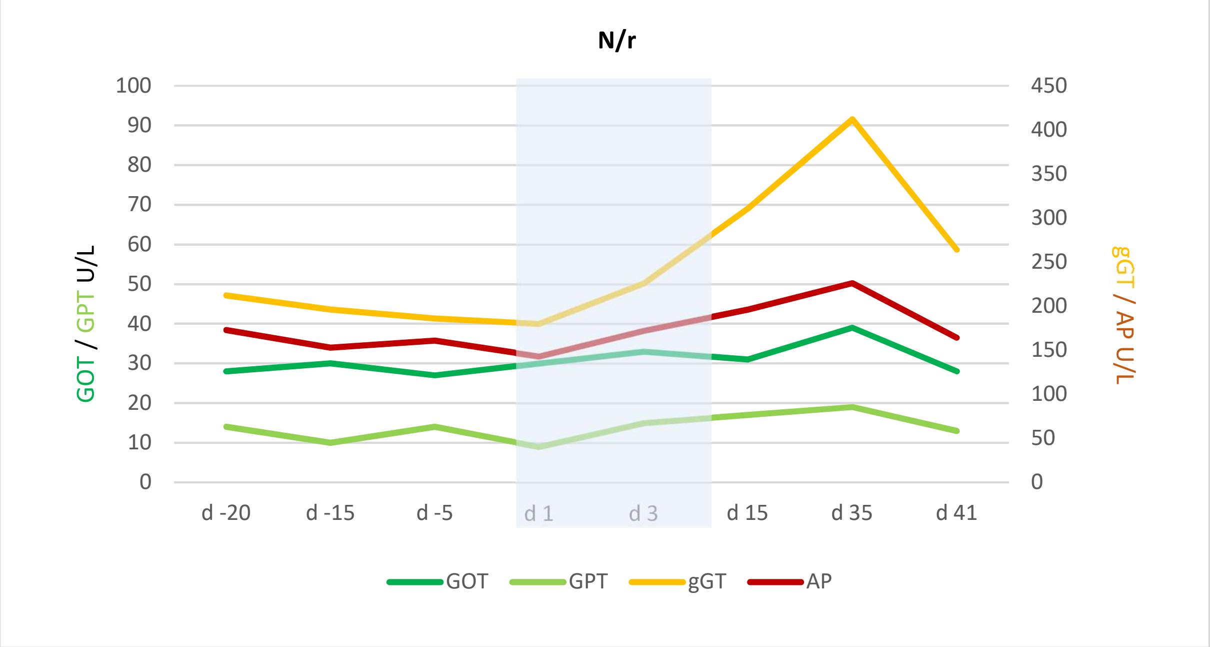

GOT: glutamic-oxaloacetic transaminase (upper limit of normal (ULN): <50U/L); GPT: glutamic pyruvic transaminase (ULN: <41U/L); GGT: Gamma-glutamyltransferase (ULN: <61U/L); AP: alkaline phosphatase (ULN: <130U/L). Grey area depicts interval of treatment with N/r (nirmatrelvir/ritonavir)

Table S1: Comparison of nirmatrelvir concentrations measured from a blood sample and either centrifuged as serum or EDTA plasma

**Timepoint (days of treatment, start = D1)**	**Concentration Nirmatrelvir ng/mL in Plasma**	**Concentration Nirmatrelvir ng/mL in Serum**	**Accuracy (%) Plasma / Serum**
**A: Patient #1**			
D3 pre-HD	-	7745	-
D3 post-HD	3636	3826	95.0
D5 pre-HD	4601	4826	95.3
D5 post-HD	2518	2729	92.3
D8 pre-HD	232.8	255.8	91.0
D8 post-HD	197.2	203.2	97.0
D10 pre-HD	39.80	38.58	103.2
D10 post-HD	26.29	31.2	84.3
D12 pre-HD	<LLOQ	<LLOQ	-
D12 post-HD	<LLOQ	<LLOQ	-
**B: Patient #2**			
D3 pre-HD	4563	4560	100.1
D3 post-HD	5765	5314	108.5
D5 pre-HD	7116	7150	99.5
D5 post-HD	5521	6630	83.3
D8 pre-HD	437.8	437.8	100.0
D8 post-HD	29.04	29.06	99.9
**C: Patient #3**			
D3 pre-HD	7898	8596	91.9
D3 post-HD	7345	7411	99.1
D5 pre-HD	6653	6675	99.7
D5 post-HD	6417	6086	105.4
D8 pre-HD	364.7	342.7	106.4
D8 post-HD	29.97	30.47	98.4
**D: Patient #4**			
D3 pre-HD	3704	3778	98,0
D3 post-HD	2308	2572	89,8
D6 pre-HD	187.3	195.2	96,0
D6 post-HD	46.91	44.97	104,3
D8 pre-HD	<LLOQ	<LLOQ	-
D8 post-HD	<LLOQ	<LLOQ	-
D10 pre-HD	<LLOQ	<LLOQ	-

Lower Limit of Quantitation (LLOQ)

Table S2: measurements dialysate of nirmatrelvir and ritonavir

Timepoint (days of treatment, start = D1)	Concentration Nirmatrelvir ng/mL in Dialysate	Concentration Ritonavir ng/mL in Dialysate
**A: Patient #1**		
D3 30min HD	254.0	<LLOQ
D5 30min HD	489.0	<LLOQ
**B: Patient #2**		
D3 30min	372.5	<LLOQ
D3 2h	380.0	<LLOQ
D3 4h	384.3	<LLOQ
D5 30min	554.6	<LLOQ
D5 2h	519.4	<LLOQ
D5 4h	442.7	<LLOQ
D8 30min	<LLOQ	<LLOQ
D8 2h	<LLOQ	<LLOQ
D8 4h	<LLOQ	<LLOQ
**C: Patient #3**		
D3 30min	783.7	<LLOQ
D3 2h	737.8	<LLOQ
D3 4h	475.4	<LLOQ
D5 30min	622.4	<LLOQ
D5 2h	466.1	<LLOQ
D5 4h	370.9	<LLOQ
D8 30min	37.3	<LLOQ
D8 2h	29.86	<LLOQ
D8 4h	28.08	<LLOQ

Lower Limit of Quantitation (LLOQ)

Table S3: Comparison of ritonavir concentrations measured from a blood sample and either centrifuged as serum or EDTA plasma

Timepoint (after treatment start = D1)	Concentration Ritonavir ng/mL in Plasma	Concentration Ritonavir ng/mL in Serum	Accuracy (%) Plasma / Serum
**A: Patient #1**			
D3 pre-HD	-	62.3	-
D3 post-HD	33.1	35.1	94.2
D5 pre-HD	30.3	31.1	97.6
D5 post-HD	14.4	16.7	86.2
D8 pre-HD	120.2	123.2	97.6
D8 post-HD	79.7	83.8	95.1
D10 pre-HD	53.4	52.7	101.4
D10 post-HD	34.7	35.3	98.2
D12 pre-HD	72.0	76.8	-
D12 post-HD	51.1	52.3	-
**B: Patient #2**			
D3 pre-HD	98.72	115.3	85.6
D3 post-HD	451.9	446.7	101.2
D5 pre-HD	553.4	613.7	90.2
D5 post-HD	527.2	534.1	98.7
D8 pre-HD	<LLOQ	<LLOQ	-
D8 post-HD	<LLOQ	<LLOQ	-
**C: Patient #3**			
D3 pre-HD	667.5	617.9	108.0
D3 post-HD	756.1	667.2	113.3
D5 pre-HD	250.0	225.9	110.7
D5 post-HD	483.6	428.8	112.8
D8 pre-HD	<LLOQ	<LLOQ	-
D8 post-HD	<LLOQ	<LLOQ	-
**D: Patient #4**			
D3 pre-HD	63.4	61.0	103.8
D3 post-HD	46.9	48.6	96.4
D6 pre-HD	<LLOQ	<LLOQ	-
D6 post-HD	<LLOQ	<LLOQ	-
D8 pre-HD	<LLOQ	<LLOQ	-
D8 post-HD	<LLOQ	<LLOQ	-
D10 pre-HD	<LLOQ	<LLOQ	-

Lower Limit of Quantitation (LLOQ)
